# Association between 9-month isoniazid prophylaxis of latent tuberculosis and severe hepatitis in patients treated with TNF inhibitors

**DOI:** 10.1038/s41598-021-97444-8

**Published:** 2021-09-09

**Authors:** Edward Chia-Cheng Lai, Hsun-Yin Liang, Ya-Chun Huang, Wei-I. Huang, Pi-Hui Chao, Wen-Wen Chen, Meng-Yu Weng

**Affiliations:** 1 School of Pharmacy, Institute of Clinical Pharmacy and Pharmaceutical Sciences, College of Medicine, National Cheng Kung University, Tainan, Taiwan; 2 Taiwan Drug Relief Foundation, Taipei, Taiwan; 3 Department of Internal Medicine, Division of Allergy, Immunology, and Rheumatology, National Cheng Kung University Hospital, College of Medicine, National Cheng Kung University, No. 138 Shen-Li Rd., Tainan, 704 Taiwan

**Keywords:** Health care, Rheumatology, Drug safety

## Abstract

To investigate associations between isoniazid for latent tuberculosis and risk of severe hepatitis, affecting patients with rheumatoid arthritis or ankylosing spondylitis whose treatment includes tumor necrosis factor inhibitors. Our self-controlled case series study analyzed Taiwan’s National Health Insurance Database from 2003 to 2015 to identify RA or AS patients, aged ≥ 20 years, receiving TNF inhibitors and a 9-month single isoniazid treatment. The outcome of interest was hospitalization due to severe hepatitis. We defined risk periods by isoniazid exposure (days): 1–28, 29–56, 57–84, 85–168, 169–252, and 253–280. To compare risk of severe hepatitis in exposed and non-exposed periods, we performed conditional Poisson regressions to generate incidence rate ratios (IRR) and 95% confidence intervals, with adjustment of patients’ baseline covariates including age, sex, HBV, HCV and related medication. Of 54,267 RA patients and 137,889 AS patients identified between 2000 and 2015, 11,221 (20.7%) RA and 4,208 (3.1%) AS patients underwent TNFi therapy, with 722 (5%) receiving isoniazid for latent tuberculosis. We identified 31 incident cases (4.3%) of hospitalization due to severe hepatitis. Of these hospitalization events, 5 occurred in the exposed periods, 25 occurred in the INH unexposed periods, and 1 occurred in the pre-exposure period. Compared with non-exposure, the risk of severe hepatitis was higher in exposed periods (incidence rate ratio [IRR]: 5.1, 95% CI: 1.57–16.55), especially 57–84 days (IRR: 17.29, 95% CI: 3.11–96.25) and 85–168 days (IRR:10.55, 95% CI: 1.90–58.51). The INH related fatal hepatotoxicity was not identified in our study. Our findings suggest an association between risk of severe hepatitis and exposure to isoniazid in patients with RA or AS under TNFi therapy, particularly within the exposed period 57–168 days. A close monitoring of liver function is mandatory to minimize the risk, especially within the first 6 months after initiation of 9 months isoniazid.

## Introduction

Rheumatoid arthritis (RA) and ankylosing spondylitis (AS) are chronic inflammatory rheumatic diseases involving peripheral joints and the axial skeleton. If they are left untreated or are unresponsive to therapy, progressive joint destruction may lead to loss of physical function, inability to perform activities of daily living, and difficulties in maintaining employment. It is widely accepted that clinical remission is the main therapeutic target for patients with RA, and a treat-to-target (T2T) strategy should be applied when treating patients with RA^1,2^. Therapeutic targets of T2T are still emerging for AS^3^. Tumor necrosis factor inhibitors (TNFi) are the first class of biological DMARDs (bDMARDs) used in treating RA and AS, their anti-inflammatory effect mainly resulting from the binding of cytokines in order to block TNF-mediated signaling^4^.

Both randomized controlled trials (RCTs) and real-world studies^5,6^ have implicated tumor necrosis factor inhibitors (TNFis) as increasing the risk of tuberculosis (TB), particularly in patients with a pre-existing latent tuberculosis infection (LTBI). Recent studies specifically demonstrate that RA patients receiving TNFi treatment are exposed to an increased risk of tuberculosis (TB) infection^7–11^. A risk management plan (RMP) for TNFi has been implemented in Taiwan since 2012, focusing on minimizing the risks of TB and hepatitis B/C virus infection. The RMP recommends that isoniazid treatment for latent TB infection (LTBI) should be given one month before TNFi therapy. The annual incidence rate of TB during TNFi treatment increased from 2008 to 2012 but has decreased since the year of RMP implementation^12^. The recommendation is for nine months of isoniazid (9H) prophylaxis in patients with LTBI who receive TNFi to prevent TB reactivation^13,14^.

Isoniazid is the most commonly used medication for LTBI in Taiwan. Nine months of isoniazid (9H) is often associated with minor, transient and asymptomatic elevations in serum aminotransferase levels. However, serious adverse events with INH for prevention of LTBI are common in primary care^15^. The long therapeutic period of isoniazid raised safety concerns regarding potentially serious or fatal hepatotoxicity^16^ and it is often asymptomatic. Although previous studies have provided information on this issue and investigated the risk of hepatitis in those receiving isoniazid for LTBI, patients with RA/AS represent a relatively small fraction of the cohorts in those studies^17–20^. In addition, many studies were subject to uncontrolled, unmeasured confounding factors, including patients’ genetic and family history, consumption of alcohol, underlying liver disease, and concurrently administered hepatotoxic drugs^21,22^. For example, Campbell et al. found that hepatotoxicity of INH was most common among patients with renal failure or use of TNFi. However, those results were based on only 64 participants completing 9H treatment^19^. Therefore, we used a population-based data to evaluate the association between risk of severe hepatitis and 9H for LTBI in patients with RA or AS under TNFi treatment. A self-controlled design was implemented to eliminate time-constant confounders.

## Materials and methods

### Study design and population

This is a population-based study using the National Health Insurance Database (NHID) of Taiwan. Taiwan has maintained a single-payer National Health Insurance (NHI) program since March 1st 1995, with 99.9% of Taiwan’s population (about 25 million individuals) enrolled by 2014^23^. The NHID compiles records from out-patient and in-patient departments, and from pharmacy services. Patients with RA were eligible to apply for a catastrophic illness certificate (CIC) offering exemption from copayment, and all applications were assessed by review committee based on relevant clinical and laboratory information. Consequently, a high degree of precision is associated with CIC diagnoses. To improve the validity of the study we linked the NHID to the catastrophic illness registry, confirming diagnosis of RA among the patient group^24^. This study was approved by the Research Ethics Committee of National Taiwan University Hospital (registration number 201803030RINC).

We used a self-controlled case series (SCCS) design for this study, whereby the relative risk is based on risk estimates for periods of exposure to isoniazid versus periods of non-exposure for the same individuals. The SCCS method allowed us to eliminate time-constant confounding factors, such as patient sex, ethnicity, and genetic factors^25,26^. The SCCS included patients aged 20 years and older diagnosed with rheumatoid arthritis (RA) or ankylosing spondylitis (AS) and receiving as the first biologic a TNFi treatment of either etanercept (Anatomical Therapeutic Chemical (ATC) code L04AB01), adalimumab (ATC code L04AB04), or golimumab (ATC code L04AB06), between 2003 and 2015 under 9H with severe hepatitis. We did not include certolizumab and infliximab in the analysis because they were not available in Taiwan during the study period. The RA diagnosis was confirmed using both records of the International Classification of Disease, Ninth Revision; ICD-9-CM code 714.0 and CIC. Because patients with AS were not eligible to apply for a CIC, we confirmed the diagnosis by identifying a record of AS (ICD-9-CM code 720.0) from inpatient claims or at least 3 records of AS from out-patient claims. Following reimbursement guidelines of Taiwan’s National Health Insurance Administration, all TNFi users must pass through review by committee members; consequently, we consider diagnosis validity for both RA and AS to be very good.

### Definitions of outcome and exposure periods

Our study outcome was severe hepatitis (ICD-9-CM 570, 573.3, 573.8 and 573.9) identified from inpatient claims for the SCCS. To ensure patients were newly diagnosed with the event, patients with a history of hepatitis recorded between 2000 and 2002 were removed from the SCCS. We retrieved all medication records to identify those receiving isoniazid (ATC code J04AC01 and J04AC51) therapy while under TNFi treatment. The 9-month single isoniazid, also known as 9H, was the only LTBI treatment covered by the NHI during the study period. Not included in our study were the newer and shorter regimens of rifamycin (e.g., 3HR or 3HP) recommended by Taiwan’s CDC since 2017; 9H has remained the most commonly used regimen because of greater convenience for doctors and patients without directly observed therapy (DOT). We removed cases with any record of hepatitis (ICD-9-CM 570, 573.3, 573.8 and 573.9) within 3 years before the starting date (i.e., 01 January 2013) to ensure all cases were new for hepatitis, without any history when they entered the SCCS.

**Figure 1:**
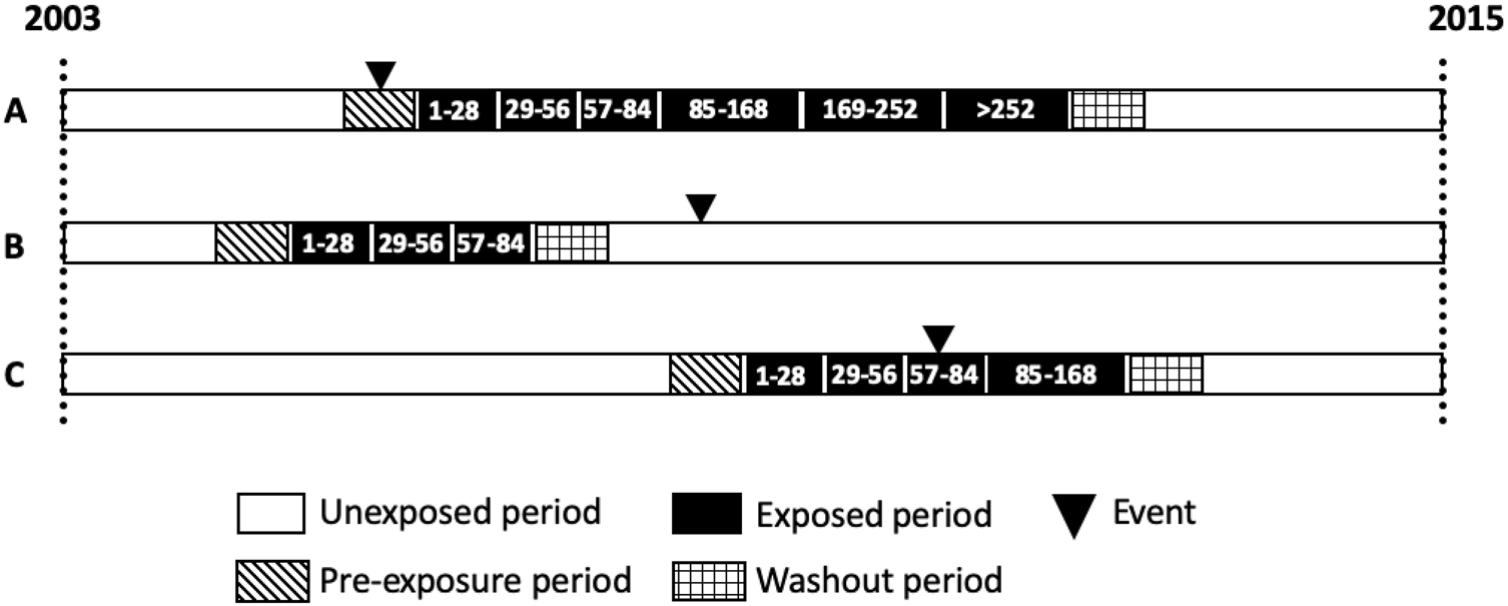
The bi-directional SCCS design of the study presenting all defined periods.

For each study participant we defined the risk periods according to duration of exposure to isoniazid. We categorized risk periods into 6 windows: 1–28 days, 29–56 days, 57–84 days, 85–168 days, 169–252 days, and 253–280 days, since most of the hepatotoxicity appeared in the first 3 months^18,19^. A 28-day pre-exposure period was added to account for the possibility that the outcome of interest may affect the likelihood of LTBI treatment, which in turn may introduce bias into the risk estimate during treatment^26^. We also designed a 28-day washout period after LTBI treatment to address the issue of residual effects of drugs after discontinuation. These two periods were analyzed independently and belong to neither exposed nor unexposed periods. All remaining time from 2003 to 2015 was considered as the unexposed period. Figure 1 presents details of risk periods within the bi-directional design of our SCCS. We captured all prescriptions of INH from the database. The periods from the prescription date plus all days when drugs were administered were considered as exposed periods. We evaluated the risk of severe hepatitis over the entire exposed period compared to unexposed periods. Moreover, we further categorized risk periods into 6 windows, to obtain more detailed risk estimates on these pre-defined exposed periods.

### Statistical analyses

Continuous variables were determined by assessing standard deviations from the mean, and categorical variables by assessing number with proportion. We calculated the incidence rate ratio (IRR) and 95% confidence interval (95% CI) by conditional Poisson regression to evaluate the risk of severe incident hepatitis associated with isoniazid in different predefined risk windows compared to the non-exposed period. Several covariates were captured during the baseline period (i.e., within 3 years before the starting date) and were considered in the regression model for risk estimation adjustments, including patients’ age, sex, HBV, HCV and co-prescribed traditional DMARD (methotrexate, sulfasalazine, hydroxychloroquine, and leflunomide), selective COX-2 inhibitors, and non-selective COX inhibitors. Besides, we performed an additional analysis by logistic regression modeling to obtain odds ratios and 95% confidence intervals, to evaluate the association between these covariates and the outcome of severe hepatitis within the cohort of 722 patients receiving 9H.

To evaluate the effects for patients who died and could not be observed in terms of further exposure to isoniazid, we conducted a sensitivity analysis by restricting our scope to surviving patients within the study period, then repeating the analysis. We used SAS version 9.4 for all statistical analyses.

### Ethics approval

All data in the study was anonymous. This study was approved by the Research Ethics Committee of National Taiwan University Hospital (registration number, 201803030RINC), Taipei, Taiwan.

## Results

### Patient characteristics of severe hepatitis

**Figure 2:**
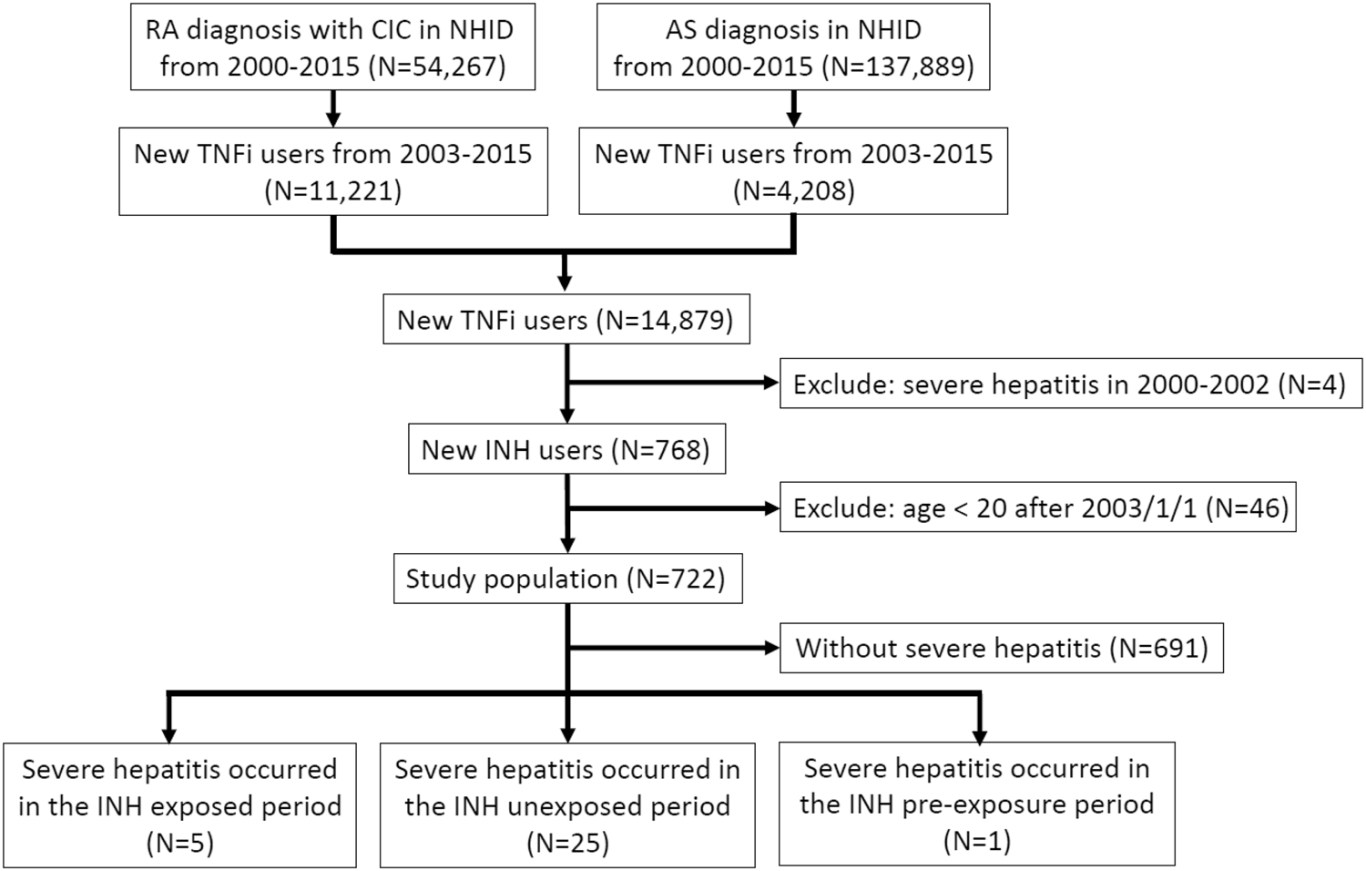
Flow diagram showing patient population of cohort selection.

We identified a total of 54,267 patients with RA and 137,889 patients with AS between 2000 and 2015, of which 11,221 (20.7%) RA patients and 4,208 (3.1%) AS patients initiated TNFi therapy. In total, 768 (5.2%) individuals initiated isoniazid LTBI treatment. We excluded 46 patients under 20 years of age (Fig. 2). The baseline characteristics of the hospitalized severe hepatitis patients are listed in Table 1. In brief, the mean age of patients was 56.15 years (SD 14.95) and 51.61% were female. We identified 6 patients (19.35%) with HBV and 5 (16.13%) with HCV. The most frequently used conventional DMARD was methotrexate (61.29%). There were 13 (41.94%), 10 (32.26%) and 4 (12.9%) patients treated with adalimumab, etanercept and golimumab, respectively (Table 1).

**Table 1:** Characteristics of TNFi users with RA or AS receiving isoniazid treatment with and without severe hepatitis.

	Severe hepatitis N (%)	No severe hepatitis N (%)	Overall N (%)
Number of patients	31	691	722
Age, mean (SD)	56.15 (14.95)	54.33 (11.83)	54.41 (11.98)
Sex, n (%)			
Female	16 (51.61)	411 (59.48)	427 (59.14)
Male	15 (48.39)	280 (40.52)	295 (40.86)
Comorbidities, n (%)			
HBV	6 (19.35)	60 (8.68)	66 (9.14)
HCV	5 (16.13)	40 (5.79)	45 (6.23)
DMARDs, n (%)			
Selective COX-2 inhibitors	25 (80.65)	502 (72.65)	527 (72.99)
Non-selective COX inhibitors	17 (54.84)	433 (62.66)	450 (62.33)
Methotrexate	19 (61.29)	427 (61.79)	446 (61.77)
Sulfasalazine	17 (54.84)	426 (61.65)	443 (61.36)
Hydroxychloroquine	14 (45.16)	302 (43.70)	316 (43.77)
Leflunomide	5 (16.13)	134 (19.39)	139 (19.25)
Etanercept	10 (32.26)	262 (37.92)	272 (37.67)
Adalimumab	13 (41.94)	302 (43.70)	315 (43.63)
Golimumab	4 (12.90)	85 (12.30)	89 (12.33)

Comorbidities (HBV, HCV) were defined as diagnosed between 2003 and 2015. Co-medications were defined as co-prescription with INH during same period. Severe hepatitis: the diagnosis of the inpatient claims data with ICD-9-CM 570, 573.3, 573.8, 573.9.

### Self-controlled case series

Table 2 presents SCCS outcomes as an evaluation of associations between isoniazid and the risk of severe hepatitis. We identified a total of 722 patients receiving 9H LTBI treatment and TNFi therapy, of whom 31 (4.29%) experienced hospitalization events for severe hepatitis. These 31 patients entered our SCCS. Of these hospitalization events, 5 occurred in the exposed periods, 25 occurred in the INH unexposed periods, and 1 occurred in the pre-exposure period. The incidence rates of severe hepatitis were 27.88 and 6.70 per 100 person-years, for exposed and unexposed periods respectively. Incidence rate ratios (IRR) were adjusted for covariates including age, sex, HBV, HCV and related medication, as shown in Table 3. Compared with the unexposed period, the risk of severe hepatitis was significantly higher in the exposed period (incidence rate ratios [IRR]:5.1, 95% CI: 1.57–16.55) of isoniazid treatment. Specifically, the IRRs for severe hepatitis were 4.00 (95% CI: 0.48–33.30), 17.29 (95% CI: 3.11–96.25), and 10.55 (95% CI: 1.90–58.51) for the exposed periods of 1–28, 57–84 and 85–168 days, respectively. The study found no instances of death due to hepatotoxicity related to INH, since any cases of death occurred during unexposed periods.

**Table 2:**
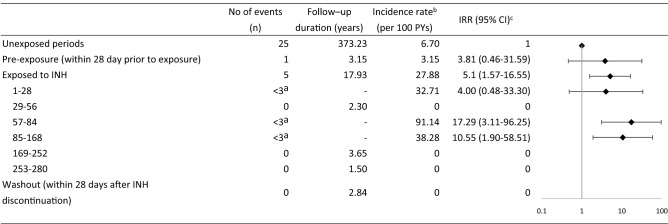
Risk of severe hepatitis before and after treatment with isoniazid (n = 31).

^a^Numbers less than 3 considered as identifiable.

^b^Incidence rate was reported as per 100 person-years.

^c^Incidence rate ratios (IRR) were adjusted for covariates listed in Table 1.

**Table 3:** Evaluation of Potential Risk factors of Severe Hepatitis Adverse Events Related to Isoniazid Treatment (n = 722).

	Adjusted OR (95% CI)^a^
Age group	
20–40	1 [reference]
41–60	0.66 (0.22–1.99)
61–80	1.23 (0.37–4.07)
> 80	4.51 (0.40–50.37)
Sex	
Male	1.56 (0.68–3.58)
Severe hepatitis risks	
HBV	2.45 (0.91–6.57)
HCV	2.24 (0.77–6.51)
DMARDs	
Selective COX-2 inhibitors	1.41 (0.53–3.74)
Non-selective COX inhibitors	0.88 (0.40–1.95)
Methotrexate	1.15 (0.49–2.73)
Sulfasalazine	0.75 (0.36–1.58)
Hydroxychloroquine	1.07 (0.47–2.46)
Leflunomide	0.87 (0.31–2.42)
Etanercept	0.47 (0.14–1.61)
Adalimumab	0.55 (0.17–1.77)
Golimumab	0.53 (0.12–2.30)

^a^Odds ratios (OR) were adjusted for all variables included in multivariate logistic regression model as shown in the table.

Table 3 presents the evaluation of risk factors for severe hepatitis. We found potential links between an increased risk of severe hepatitis and age (in older patients), male sex, HBV, HCV, and use of selective COX-2 inhibitors or methotrexate, although this lacked significant statistical support. There were 4 patients who died during the unexposed period; these patients were removed following sensitivity analysis, as indicated in Table 4. Compared with the unexposed period, the risk of severe hepatitis was still significantly higher in the exposed period of isoniazid treatment (incidence rate ratios [IRR]: 4.75, 95% CI: 1.43–15.86). The IRRs for severe hepatitis were 3.15 (95% CI: 0.37–26.46), 14.64 (95% CI: 2.49–86.04), and 9.41 (95% CI: 1.68–52.62) for the exposed periods of 1–28, 57–84 and 85–168 days, respectively. The sensitivity analysis (restricting our study to surviving patients) showed results consistent with the main analysis.

**Table 4:**
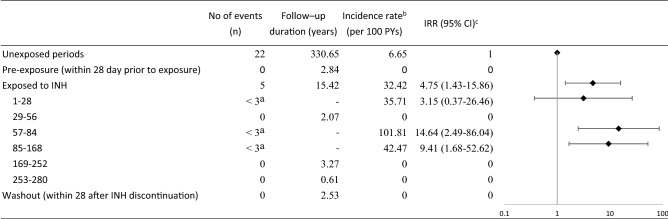
Sensitivity analysis of risk of severe hepatitis before and after treatment with isoniazid (excluding death cases) (n = 27).

^a^Numbers less than 3 considered as identifiable.

^b^Incidence rate was reported as per 100 person-years.

^c^Incidence rate ratios (IRR) were adjusted for covariates listed in Table 1.

## Discussion

We found the use of isoniazid for LTBI treatment was associated with an increased risk of severe hepatitis in patients with RA or AS undergoing TNFi therapy. The risk was especially high between 57 and 168 days after isoniazid initiation. There were 5 cases of hospitalization due to severe hepatitis among the 722 persons during the time they were exposed to isoniazid. None of those patients died from the events. Tuberculosis is a leading cause of death worldwide, with 10.0 million cases and 1.4 million deaths in 2019^27^. Patients treated with TNFi are at moderate risk of latent tuberculosis reactivation^28^. The reasons for frequent reactivation of LTBI in patients with inflammatory arthritis are complex but include the disease itself, use of DMARDs and steroids. Currently and in multiple countries, INH treatment of LTBI remains the most commonly used medication to prevent TB reactivation^17,29^. However, isoniazid treatment for LTBI is under debate mainly because of continuing concerns about hepatotoxicity. The risk and results of isoniazid hepatotoxicity may influence preventive therapy. Multiple RCTs have demonstrated that rifamycin-based treatment regimens have lower rates of hepatotoxicity, better completion, and non-inferior efficacy in treating LTBI^30–32^.

Although severe hepatitis has been identified as a known side effect of isoniazid, the risk profile has not been well-evaluated in patients with RA and AS who undergo TNFi therapy. In early studies of general population, the rate of hepatotoxicity due to isoniazid for LTBI treatment was 0.1% to 3.0%^33–37^. In RA patients, the frequency of hepatotoxicity is much higher, with one study showing that 12.5% of RA patients treated with isoniazid experienced liver function abnormalities while using TNFi^38^. In an American single center study, 11% of RA patients treated with both methotrexate and isoniazid had transient increased liver function in test results^39^. A study observed 9H-related hepatotoxicity in RA patients concomitantly treated with methotrexate or sulfasalazine during TNFi treatment; among a total of 8 patients receiving isoniazid treatment, 4 developed liver dysfunction^40^. In a multicenter randomized controlled trial in Taiwan, discontinuation rates owing to adverse drug reactions were 5.3% in patients treated with 9H, the study identifying 7 patients who had developed clinically relevant hepatotoxicity^41^. However, the relatively small sample size of TNFi users and rare incidence of severe hepatitis events in these studies should be taken into account. Many previous studies defined isoniazid-induced serious liver injury based on American Thoracic Society (ATS) criteria of grade 3 or 4 events. None of the studies focus on severe hepatitis that requires hospitalization in specific groups of patients, such as RA and AS, who require polypharmacy which is itself hepatotoxic. Our results highlight the importance of close monitoring of liver function to prevent the occurrence of severe hepatic adverse events in these groups of patients.

In the biologic era, rheumatologists should take special care with the use and monitoring of long-term isoniazid treatment in combination with potentially hepatotoxic drugs such as cDMARDs and NSAIDs. RA patients in Taiwan require at least 2 conventional DMARDs with a refractory response before commencing TNFi therapy. Currently, isoniazid is the most commonly used medication for LTBI in Taiwan. It is associated with various levels of hepatitis in general population^17^. Concerning drug-drug interaction, 9H may have less effect compared with rifamycin regimens, especially for patients with RA and AS who usually require several kinds of medication. In our study the incidence rate of hospitalized severe hepatitis during isoniazid treatment was only 0.69%, a lower figure than identified in the aforementioned studies. We believe that these differences in results can be explained by different definitions of hepatotoxicity. Isoniazid-associated hepatotoxicity can be severe and indeed life threatening, thus of particular concern to clinicians. For a solid outcome that is clinically significant, we defined severe hepatitis as that which led to hospitalization, as recorded in the inpatient claims data. Consistent with a previous study^18^, the risk of hepatitis in our study was higher between 57 and 168 days after exposure to isoniazid. This finding suggests aggressive monitoring of liver function is mandatory to minimize the risk of severe hepatitis, especially in the initial 6 months of 9H treatment.

To our knowledge, compared with previous studies, this is the largest study currently including incident cases of severe hepatitis in sufficient numbers of RA and AS patients who undergo TNFi treatment while on 9H; this study allows more definite conclusions regarding the association between 9H therapy for LTBI and severe hepatitis. A significant strength of our study is that we used a large population-based database to provide sufficient sample size and statistical power for analysis. This allowed us to evaluate events with a very low incidence rate which were nonetheless clinically significant to RA or AS patients under TNFi, conventional DMARD and NSAID therapies. Moreover, we used the SCCS design to minimize the unmeasured confounders. Each patient acted as his/her own control. Despite the inclusion of many potential confounders in the multivariate models, residual confounding by factors that are not measured in claims databases may remain. The SCCS design provides relative incidence estimates in high-risk periods compared with low-risk periods^26^. This method therefore controls for time-constant confounders and also unmeasured confounders^42^.

Our study presents some limitations. First, we do not know the overall numbers of LTBI patients in these cohorts. Second, there was no laboratory data available in the claims data to which our study referred; we thus could only use inpatient records to define severe hepatitis. However, to improve the validity of diagnosis, we have only captured acute hepatitis from the diagnosis in inpatient claims data. Consequently, the actual number of cases with severe hepatitis may be an underestimate. Our analysis only included patients with severe hepatitis requiring hospitalization. No inference can be made for the association between 9H treatment and relatively mild hepatic reactions from our analysis. Third, in using the NHID, we did not have access to information regarding the patient’s alcohol consumption or self-payment medication. Forth, as with all studies using a claims database, we were unable to confirm the drug compliance of patients. However, a post-hoc analysis found the overall proportion of drugs covered (PDC) was more than 89.29% for those who did not experience any severe hepatitis during the exposed period. Generally, however, any patients discontinuing treatment would be unable to refill their prescription for 9 months. Fifth, the generalizability of this study to Western populations should be discussed since Asian populations had a higher baseline risk of hepatitis, possibly due to higher rates in Asia of hepatitis B virus (HBV) and hepatitis C virus (HCV) infection^43,44^. Consequently, HBV and HCV infection have been included in our regression models for adjustment. Because the recurrent severe hepatitis was not independent of the first occurrence, our analysis only considered the first events of severe hepatitis, as suggested by Irene Petersen et al.^45^. We may underestimate the risk of severe hepatitis for the exposed periods if patients had severe hepatitis before exposure. The analytic population in our study likely represents a lower-risk subset of the total RA/AS population and the effect estimate may be different for other groups of patients. Lastly, although we have examined a large population-based database with 23 million individuals for this study, only 31 patients entered the SCCS resulting in wide confidence intervals around the estimates.

## Conclusions

Our findings suggest an association between risk of severe hepatitis and exposure to isoniazid in patients with RA or AS, and the risk was especially high within the exposed period of 57–168 days. The findings warrant attention because liver impairment is usually asymptomatic and we may delay stopping isoniazid for those with liver impairment. A close monitoring of liver function is mandatory to minimize the risk, especially within the first 6 months after initiation of 9 months isoniazid.
