# Publisher Correction: Association between 9-month isoniazid prophylaxis of latent tuberculosis and severe hepatitis in patients treated with TNF inhibitors

**DOI:** 10.1038/s41598-021-99382-x

**Published:** 2021-10-12

**Authors:** Edward Chia‑Cheng Lai, Hsun‑Yin Liang, Ya‑Chun Huang, Wei‑I. Huang, Pi‑Hui Chao, Wen‑Wen Chen, Meng‑Yu Weng

**Affiliations:** 1 School of Pharmacy, Institute of Clinical Pharmacy and Pharmaceutical Sciences, College of Medicine, National Cheng Kung University, Tainan, Taiwan; 2 Taiwan Drug Relief Foundation, Taipei, Taiwan; 3 Department of Internal Medicine, Division of Allergy, Immunology, and Rheumatology, College of Medicine, National Cheng Kung University Hospital, National Cheng Kung University, No. 138 Shen-Li Rd, Tainan, 704 Taiwan

Correction to: *Scientific Reports* 10.1038/s41598-021-97444-8, published online 09 September 2021.

In the original version of this Article, the image on the right in Table 2 and Table 4 did not display correctly. As a result, each bar of the forest plot did not align with the IRR (95% CI) in the adjacent columns.

**Table 2:** Risk of severe hepatitis before and after treatment with isoniazid (n = 31). The figure on right hand side is skew (did not match the IRR)

	No of events (n)	Follow-up duration (years)	Incidence rate^b^ (per 100 PYs)	IRR (95% CI)^c^	
Unexposed periods	25	373.23	6.70	1	
Pre-exposure (within 28 day prior to exposure)	1	3.15	3.15	3.81 (0.46–31.59)	
Exposed to INH	5	17.93	27.88	5.1 (1.57–16.55)	
1–28	< 3^a^	–	32.71	4.00 (0.48–33.30)	
29–56	0	2.30	0	0	
57–84	< 3^a^	–	91.14	17.29 (3.11–96.25)	
85–168	< 3^a^	–	38.28	10.55 (1.90–58.51)	
169–252	0	3.65	0	0	
253–280	0	1.50	0	0	
Washout (within 28 days after INH discontinuation)	0	2.84	0	0	

^a^Numbers less than 3 considered as identifiable.

^b^Incidence rate was reported as per 100 person-years.

^c^Incidence rate ratios (IRR) were adjusted for covariates listed in Table 1.

**Table 4:** Sensitivity analysis of risk of severe hepatitis before and after treatment with isoniazid (excluding death cases) (n = 27). The figure on right side is skew (did not match the results of IRR).

	No of events (n)	Follow-up duration (years)	Incidence rate^b^ (per 100 PYs)	IRR (95% CI)^c^	
Unexposed periods	22	330.65	6.65	1	
Pre-exposure (within 28 day prior to exposure)	0	2.84	0	0	
Exposed to INH	5	15.42	32.42	4.75 (1.43–15.86)	
1–28	< 3^a^	–	35.71	3.15 (0.37–26.46)	
29–56	0	2.07	0	0	
57–84	< 3^a^	–	101.81	14.64 (2.49–86.04)	
85–168	< 3^a^	–	42.47	9.41 (1.68–52.62)	
169–252	0	3.27	0	0	
253–280	0	0.61	0	0	
Washout (within 28 after INH discontinuation)	0	2.53	0	0	

^a^Numbers less than 3 considered as identifiable.

^b^Incidence rate was reported as per 100 person-years.

^c^Incidence rate ratios (IRR) were adjusted for covariates listed in Table 1.

Additionally, legends for Table 2 and Table 4, erroneously included the sentence: “The figure on right hand side is skew (did not match the IRR)” which has now been removed.

The original Table 2 and Table 4 and their accompanying legends appear below.

The original Article has been corrected.

